# Characterizing inner-shell with spectral phase interferometry for direct electric-field reconstruction

**DOI:** 10.1038/ncomms6599

**Published:** 2014-12-16

**Authors:** Hiroki Mashiko, Tomohiko Yamaguchi, Katsuya Oguri, Akira Suda, Hideki Gotoh

**Affiliations:** 1 NTT Basic Research Laboratories, 3-1 Morinosato Wakamiya, Atsugi, Kanagawa 243-0198, Japan; 2 Tokyo University of Science, 2641 Yamazaki, Noda-shi, Chiba-ken 278-8510, Japan

## Abstract

In many atomic, molecular and solid systems, Lorentzian and Fano profiles are commonly observed in a broad research fields throughout a variety of spectroscopies. As the profile structure is related to the phase of the time-dependent dipole moment, it plays an important role in the study of quantum properties. Here we determine the dipole phase in the inner-shell transition using spectral phase interferometry for direct electric-field reconstruction (SPIDER) with isolated attosecond pulses (IAPs). In addition, we propose a scheme for pulse generation and compression by manipulating the inner-shell transition. The electromagnetic radiation generated by the transition is temporally compressed to a few femtoseconds in the extreme ultraviolet (XUV) region. The proposed pulse-compression scheme may provide an alternative route to producing attosecond pulses of light.

## Abstract (web summary)

Characterizing and understanding the profile of atomic spectral lines tells us a great deal about an atom’s properties. Here, the authors combine attosecond transient spectroscopy and the SPIDER technique to reconstruct the dipole oscillation phase of the auto-ionisation inner-shell transition in neon.

## Graphical abstract

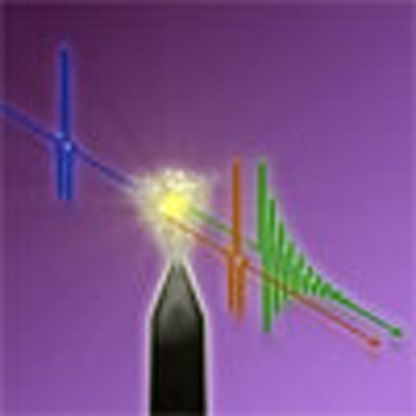

Electron–electron correlation with two-wave functions was explained by Fano in 1961 (ref. 1). The now famous symmetric Lorentzian and asymmetric Fano profiles observed in the spectral domain were explained by the interference of a discrete state with a continuum state^1^,^2^. The profile is observed in a variety of spectroscopies, such as absorption^3^, photoelectron^4^, fluorescence^5^, wave-mixing^6^ and Raman scattering^7^, which covers fields in physics, chemistry and biology throughout atoms^8^, molecules^9^ and solids^10^.

Recently, Ott *et al.*^11^ demonstrated the transformation from a symmetric Lorentzian profile to an asymmetric Fano profile by controlling the initial phase shift of the time-dependent dipole moment in a helium atom. Their work confirmed that the origin of the asymmetric Fano profile is a phase shift of the dipole oscillation induced by the transition to a discrete state with respect to that induced by the transition to a continuum state. This time-domain picture of the Fano profile shed light on the temporal response of the dipole moment, which is directly related to the quantum phase of excited states induced by an input laser field.

Generally, the dipole moment is related to an absorption cross-section, which corresponds to an imaginary part of the refractive index. From an optical point of view, the temporal dipole response corresponds to a temporal response of the electromagnetic radiation generated by the dipole oscillation. This indicates that extraction of phase information from classical electromagnetic radiation is directly connected to a determination of the quantum phase of the excited states. The Lorentzian or Fano profile observed in an absorption spectrum is an interference pattern constructed by two fields of electromagnetic radiation. The radiation is generated by the dipole oscillations in the transition to the continuum and discrete states, including their coupling. The phase of the dipole moment can be extracted with an optical pulse characterization technique based on spectral phase interferometry for direct electric-field reconstruction (SPIDER)^12^,^13^. This is our basic idea.

In recent years, isolated attosecond pulses (IAPs) have been produced by high-harmonic generation^14^,^15^,^16^,^17^ and used to study the lifetime of the inner-shell transitions with ultrafast decay^18^,^19^,^20^,^21^,^22^. The extremely short IAP with a supercontinuum spectrum, which behaves like a Dirac delta function, is also expected to be a useful reference pulse for monitoring the dipole phase in the transition to the discrete state. Here we propose reconstructing the dipole phase induced by the inner-shell transition using a SPIDER scheme. With this method, we successfully determined the phase of the dipole oscillation with autoionizing inner-shell transition in a neon atom using a temporally characterized IAP. In addition, electromagnetic radiation with temporal and spatial coherence has substantial importance in the extreme ultraviolet and X-ray regions, as optical crystals are very lacking in short-wavelength regions. Here we propose a pulse compression scheme for radiation using an intense few-cycle near-infrared (NIR) pulse. As the excited dipole oscillation in the neon target is controlled by the delay time of the NIR pulse, radiation at the resonance frequency with a controllable duration can be produced.

## Results

### Dipole phase determination with SPIDER

Autoionization peaks with Lorentzian or Fano profile structures are observed by absorption spectroscopy and are represented by Fano’s formula^1^


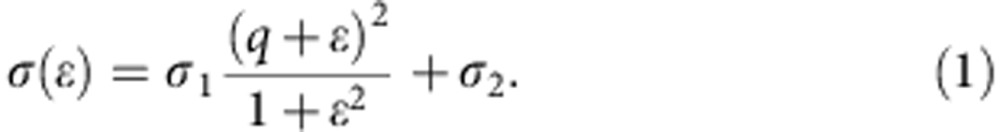


**Figure 1:**
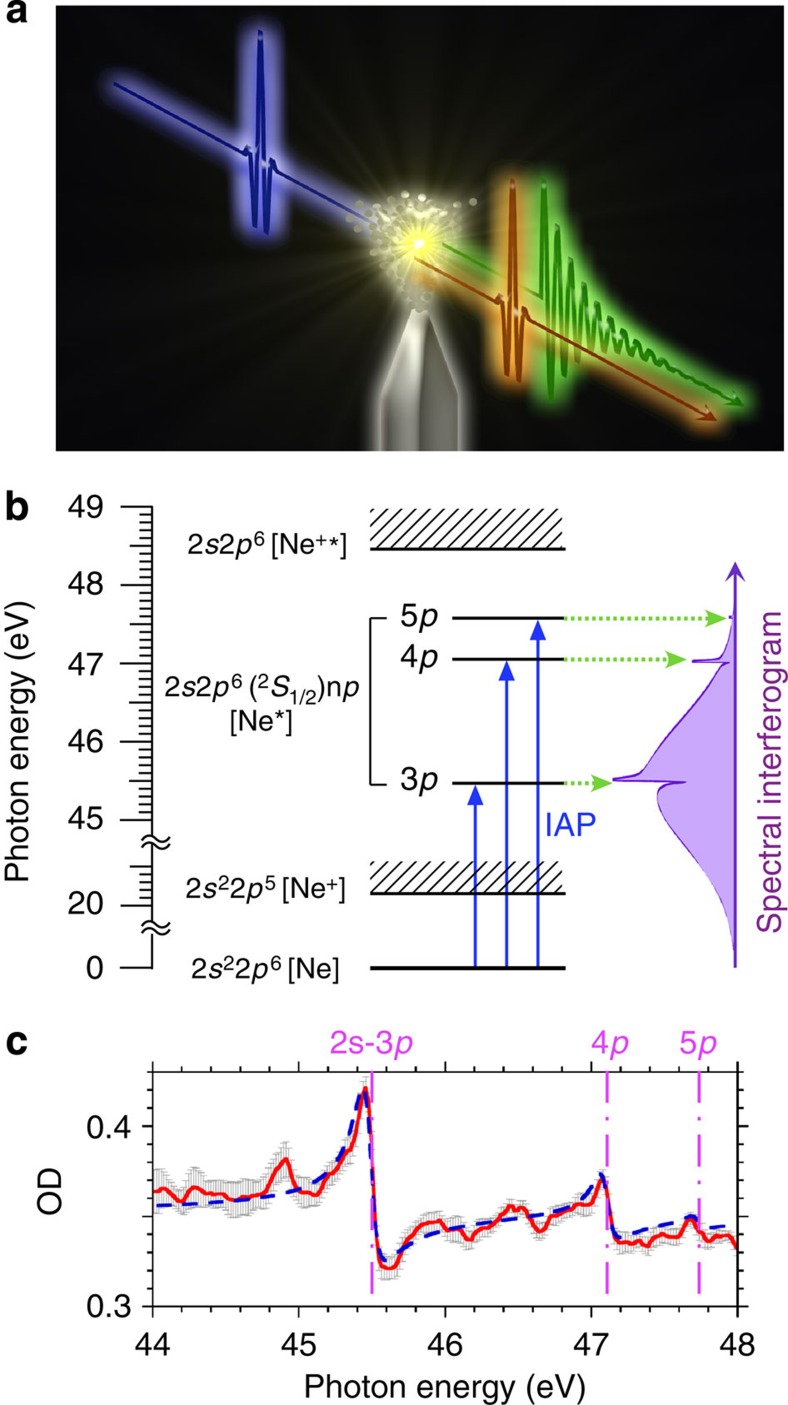
Energy level diagram and absorption spectrum. (**a**) Schematic view of the electromagnetic radiation with the dipole oscillation induced by the IAP. (**b**) Diagram of a neon atom with the 2*s*2*p*^6^(^2^*S*_1/2_)n*p* (*n*=3–5) autoionization states. The arrows indicate the IAP (blue solid) and the electromagnetic radiation from the states (green dotted). The shaded purple region is the interferogram. (**c**) The absorption spectra with measurement (red solid line) and calculation using equations (1) and (2) (blue dashed line). The error bar represents s.d. in 30 measurements. The pink dashed-solid line shows the resonance energies with autoionizing transitions from 2*s* to 3*p*, 4*p* and 5*p*.

The *σ*(*ε*) is the total absorption cross-section, whereas *σ*_1_ and *σ*_2_ represent the cross-sections corresponding to the transition to the continuum and the discrete autoionization states, respectively. The Fano line shape parameter *q* relates to the initial phase of dipole oscillation^1^,^11^. Here


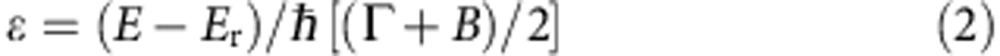


indicates the reduced energy. The incident energy *E*=*ħω* corresponds to continuum absorption in the frequency domain and instantaneous excitation in the temporal domain (ideally, the Dirac delta function). The *ω* and *ħ* are frequency and Plank’s constant, respectively. The resonance energy *E*_r_ corresponds to the transition to the discrete state. The Γ is the linewidth of the discrete state. In experimental analysis, the detector resolution *B* is the full-width at half-maximum (FWHM) for the Lorentzian function, which is added to Fano’s formula^8^. Figure 1a shows a schematic view of the electromagnetic radiation with the dipole oscillation induced by the IAP. The energy level diagram of a neon atom with Ne* 2*s*2*p*^6^(^2^*S*_1/2_)n*p* (*n*=3–5) autoionization states as shown in Fig. 1b (see ref. 23). Figure 1c shows measured (red solid line) and calculated (blue dashed line) absorption profiles with the IAP at optical density (OD) (The details of the experimental setup and the definition of OD are explained in the method section.). The properties of the inner-shell transition from Ne 2*s*^2^2*p*^6^ to Ne* 2*s*2*p*^6^(^2^*S*_1/2_)3*p* are the resonance energy *E*_r_ of 45.5 eV, the linewidth Γ of 13 meV, and the Fano line shape parameter *q* of −1.6^3^. We estimated the detector resolution *B* of 114 meV based on equations (1) and (2). The cross-section *σ*_1_ in the continuum state is 7.5 Mb at the resonance energy of 45.5 eV (ref. 4). The *σ*_2_ in the discrete autoionization state is 3.2 Mb at 45.5 eV (ref. 4). The estimated decay time and the initial phase of dipole oscillation are 35 fs at FWHM (50.6 fs at 1/e) and 0.35π (ref. 11), respectively. Regularly, the excited electron in the 3*p* state decays into Ne^+^ 2*s*^2^2*p*^5^*εs* and *εd* continuum states on the basis of the configuration interaction.

To reconstruct the dipole phase *φ*_Dis_(*ω*) in the transition to the discrete state, the formula of SPIDER is applied:^12^,^24^


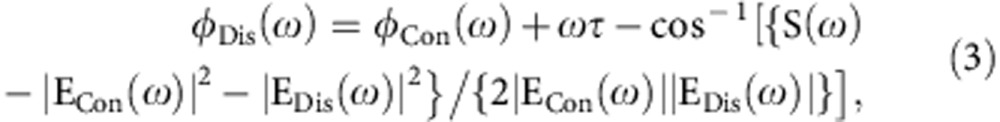


where the *ω* is laser frequency. The *φ*_Con_(*ω*) is the dipole phase in the transition to the continuum state. The *E*_Con_(*ω*) (continuum spectrum) and *E*_Dis_(*ω*) (Lorentzian spectrum) are the amplitudes of the electromagnetic radiation generated from the target medium with the transition to the continuum and the discrete autoionization states, respectively. The amplitude corresponds to the strength of the dipole. The *S*(*ω*) is the spectral interferogram produced by both transitions. The *τ* is delay time between the *E*_Con_(*t*) (ideally, the Dirac delta function) and the *E*_Dis_(*t*) (exponential decay function) in the temporal domain. To determine phase *φ*_Dis_(*ω*), first phase *φ*_Con_(*ω*) has to be known. In this experiment, we approximated that dipole phase *φ*_Con_(*ω*) with the transition to the continuum state is a replica of the spectral phase of the input IAP used for the excitation. This approximation for the transition to the continuum state is similar to the most common methods for attosecond pulse characterization: FROG-CRAB (frequency-resolved optical gating for complete reconstruction of attosecond bursts)^25^ and RABITT (reconstruction of attosecond beating by interference of two-photon transitions)^26^.

**Figure 2:**
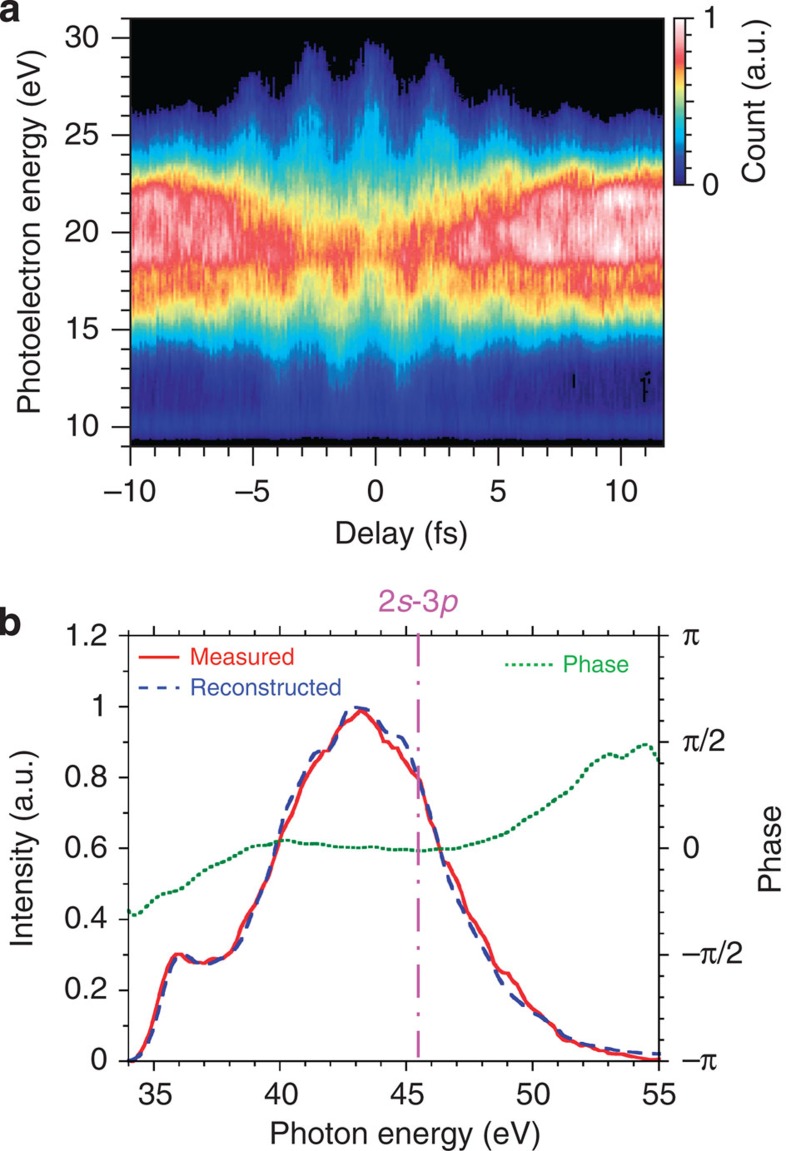
Temporal characterization of IAP. (**a**) The measured attosecond streak trace with a helium atom. (**b**) The reconstructed spectrum (blue dashed line) and phase (green dotted line). For comparison, the measured spectrum (red solid line) without the streak field of NIR pulse is also shown. The pink dashed-solid line corresponds to the resonance energy with the Ne 2*s*-3*p* transition.

**Figure 3:**
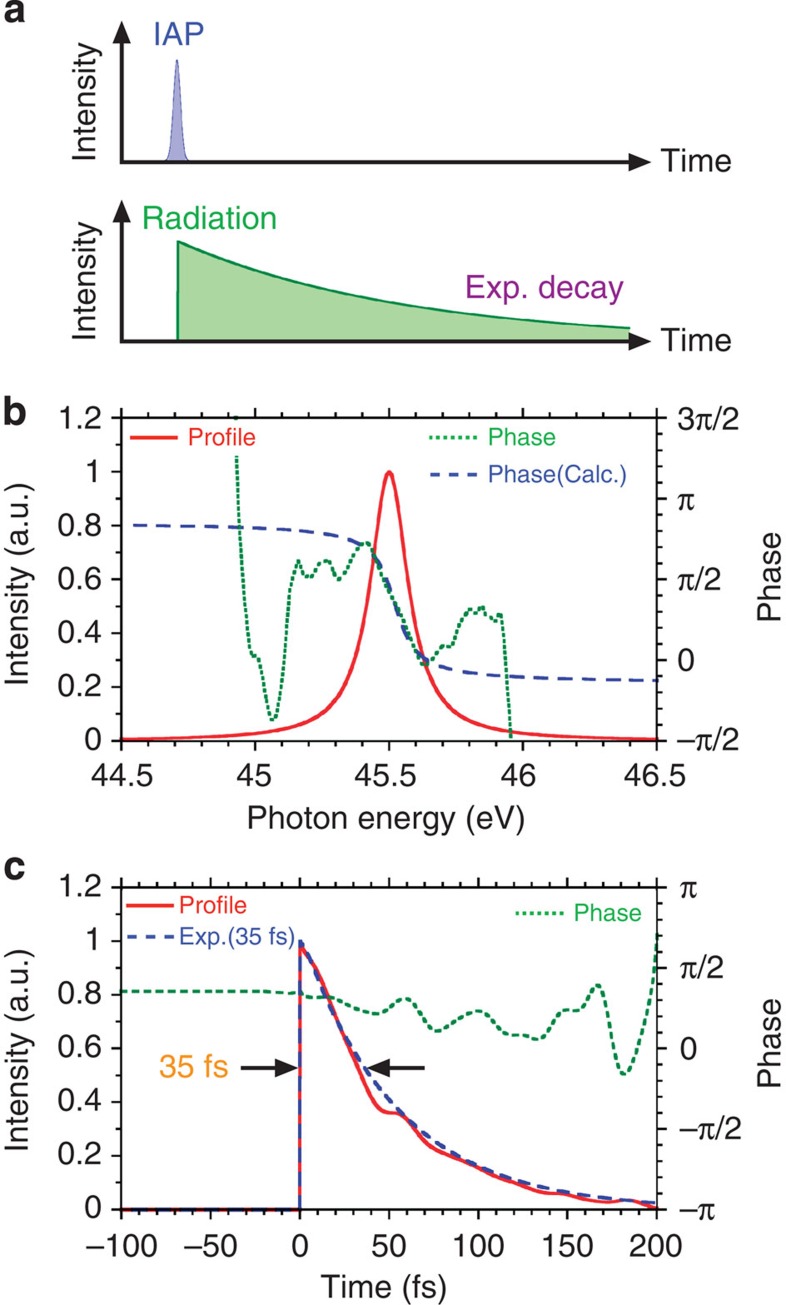
Schematic view of the temporal interaction and phase reconstructions. (**a**) The interaction with the IAP (blue shaded) and the electromagnetic radiation in Ne 2*s*-3*p* transition with the exponential decay curve (green shaded). (**b**) The spectrum (red solid line) and the phases in reconstruction (green dotted line) and calculation (blue dashed line). (**c**) The temporal profiles in reconstruction (red solid line) and calculation with 35-fs decay time (blue dashed line). The green dotted line shows the reconstructed phase.

Figure 2a shows a typical attosecond streak trace using a helium atom. The experimental condition is described in the Methods section. The IAP spectrum reconstructed by the FROG-CRAB method (blue dashed line) agrees well with measured spectrum (red solid line), as shown in Fig. 2b. The reconstructed phase (green dotted line) is nearly flat at the energy of 45.5 eV, which corresponds to the Ne 2*s*-3*p* transition. The reconstructed temporal profile indicates a 192-as duration. The characterized IAP is applied for the neon atom in absorption spectroscopy. Figure 3a shows a schematic view of the IAP (blue shaded) and the electromagnetic radiation via the autoionizing transition (green shaded) in temporal domain. With the extremely short IAP, the electromagnetic radiation has an exponential decay curve in the temporal domain. In the spectral domain, it has a Lorentzian profile at Fourier transformation. Figure 3b shows the estimated spectrum (red solid line) and the reconstructed (green dotted line) and calculated (blue dashed line) spectral phases (the Methods section describes how the spectrum is estimated). To obtain the statistics, we accumulated 20,000 laser shots. The calculated phase is simply derived from the exponential function with decay time of 35 fs at the FWHM, and it agrees well with the reconstructed phase at near the centre portion. The large phase error in the edge of the Lorentzian (Fano) profile is mainly caused by the insufficient signal-to-noise ratio in the experimental detection. When the amplitude of the Fano profile approaches to the amplitude of detection noise, the interferometric visibility constructed with the Fano profile is reduced. Then, the error of reconstructed phase is increased. Figure 3c shows the reconstructed temporal profile (red solid line) and phase (green dotted line). The reconstructed temporal profile nearly agrees with the exponential decay curve with 35-fs duration (blue dashed line). Principally, this method allows us to reconstruct the phase with single-laser-shot measurement, which is useful for real-time monitoring of various transitions.

### Pulse compression with transition manipulation

**Figure 4:**
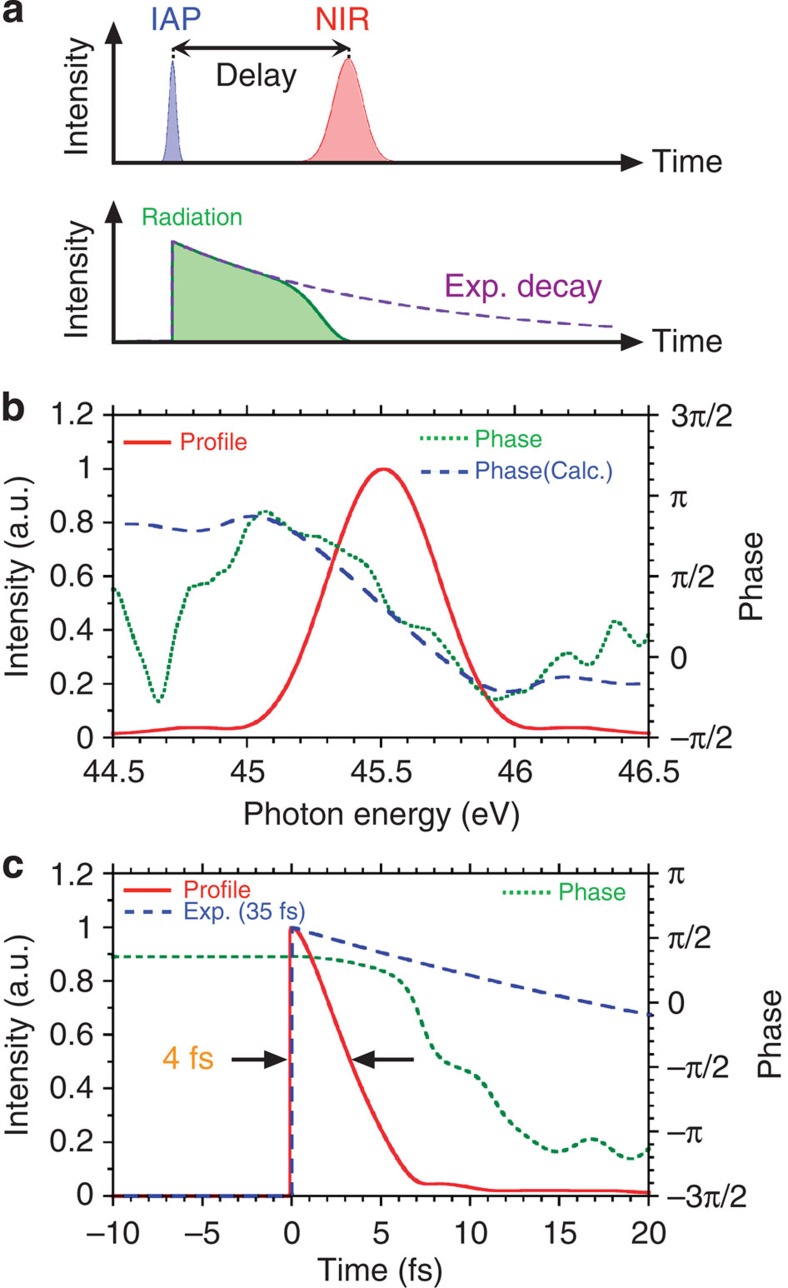
Schematic view of the temporal interaction and phase reconstructions. (**a**) The interaction with the IAP (blue shaded), NIR pulse (red shaded) and the electromagnetic radiation in Ne 2*s*-3*p* transition (green shaded). (**b**) The spectrum (red solid line) and the phases in reconstruction (green dotted line) and calculation (blue dashed line) corresponding to 10-fs delay time between the IAP (first pulse) and NIR pulse (second pulse). (**c**) The temporal profiles in reconstruction (red solid line) and calculation with 35-fs decay time (blue dashed line). The green dotted line shows the reconstructed phase.

Meanwhile, electromagnetic radiation with temporal and spatial coherence has a large capability to generate ultrashort pulses in the extreme ultraviolet and X-ray regions. When the autoionization state is dressed by the NIR pulse (with photon energy centered at 1.65 eV), the electron is excited to the Ne^+*^ 2*s*2*p*^6^ continuum state (above 48.5 eV) via the two-photon process. Thus, the NIR pulse tends to truncate the exponential decay curve in the temporal domain as shown in Fig. 4a. As the pulse duration of the electromagnetic radiation is shorter than the exponential decay, the spectrum should be broader than the original linewidth in the Lorentzian profile. Note, in this Ne 2*s*-3*p* transition, another discrete state satisfying the dipole selection rule does not exist near the state within 10 photons of the NIR pulse^23^. This avoids the obvious spectral split and shift with the AC Stark effect near the autoionization peak^27^. Figure 4b shows the spectrum (red solid line) and the spectral phase (green dotted line) corresponding to 10-fs delay time between the IAP (first pulse) and NIR pulse (second pulse). The blue dashed line shows the simulated spectral phase derived from the truncated exponential decay curve in the temporal domain. Figure 4c shows the reconstructed temporal profile (red solid line) and phase (green dotted line). The blue dashed line is the exponential decay curve with 35-fs duration. Consequently, the duration is temporally compressed to 4 fs with the transition manipulation.

**Figure 5:**
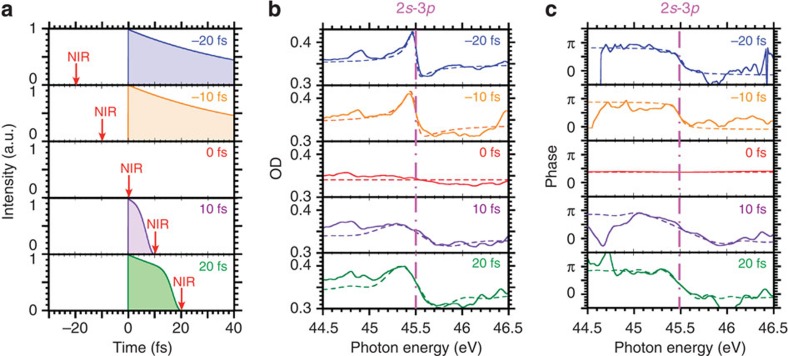
Transient OD traces and the phases in each delay times. (**a**) Schematic view of the temporal profiles of the electromagnetic radiation at the delay times of −20 (upper), −10, 0, 10 and 20 fs (bottom). The red arrow corresponds to input timing of NIR pulse. (**b**) The OD traces in measurement (solid lines) and calculation (dashed lines) at each delay time. (**c**) The phases in reconstruction (solid lines) and calculation (dashed lines) with each delay time. The pink dashed-solid lines correspond to the resonance energy in Ne 2*s*-3*p* transition.

Furthermore, this method allows us to reconstruct the phase evolution at each delay time between the IAP and NIR pulse. Figure 5a shows a schematic view of the temporal interactions at delay times of −20, −10, 0, 10 and 20 fs. Figure 5b, c shows measured and calculated OD profiles and the reconstructed and calculated phases, respectively. While the IAP (first pulse) has large delay from the NIR (second pulse), the profile of the autoionization peak is not modified because the NIR pulse cannot dress the autoionization state. When the IAP and the NIR pulse are temporally overlapped, the spectrum is broadened. When the NIR pulse (first pulse) has large delay from the IAP (second pulse), the broadened spectrum gradually becomes narrow again because the truncation effect for exponential decay curve with IAP depends on the decay time of the autoionization state. For future application, if the intense NIR pulse sharply truncates the exponential decay curve with half-cycle temporal gate depending on the carrier-envelope phase, the electromagnetic radiation can be temporally compressed further to attosecond duration. As the radiation with polarizability also involves the wavevector, it can be spatially isolated^11^,^28^. If an X-ray free electron laser is the pump-pulse, the flux will be increased, even in the hard X-ray region^29^. As the conversion efficiency is basically related to the cross-section value, it may be higher than in high-harmonic generation in the shorter wavelength region.

## Discussion

We demonstrated the determination of the phase in time-dependent dipole oscillation in the inner-shell transition in a neon atom using the combination of attosecond transient spectroscopy and SPIDER. Principally, the combination scheme allows us to monitor the phase in a single-laser-shot measurement and is useful for real-time observation. Thus, it is powerful for understanding the quantum properties and for application to attosecond coherent control for the inner-shell transition in future. In addition, the electromagnetic radiation generated by the transition is temporally compressed to 4 fs from 35 fs with the transition manipulation using an extra few-cycle NIR pulse. This scheme has the capability to generate another type of IAP and will be a valuable additional technology in both ultrashort pulse generation and attosecond science fields.

## Methods

### Transient absorption spectroscopy

A Ti:sapphire-laser-based few-cycle NIR pulse with 7-fs duration and centre photon energy of 1.65 eV was used for high-harmonic generation and the probe-pulse for the transient absorption^30^. The details of the pump–probe system are described in ref. 31. In this system, the pump–probe timing jitter is 20 as at the root mean square measured over 12 h. To generate the IAP, we used a double optical gating^32^ technique for high-harmonic generation with argon gas. The IAP passes through an aluminum filter with 300 nm thickness. The IAP and NIR pulse are collinearly propagated and focused to a neon gas-filled cell (500-μm interaction length; estimated pressure of 30 mbar) with a Mo/Si mirror^33^. The estimated target intensity of the NIR pulse is ~2 × 10^12^ W cm^−2^. After the cell, the electromagnetic radiation generated by the neon atom is sent to a photon spectrometer. The spectral resolution is 114 meV at 45.5 eV photon energy.

### Definition of OD

In this experiment, we defined the OD at laser frequency *ω* as OD(*ω*)=−log[*|E*_in_(*ω*)|^2^/*|E*_out_(*ω*)|^2^], where *E*_in_(*ω*) is the electric-field strength of input IAP. The *E*_out_(*ω*) corresponds to the electromagnetic radiation generated by a neon atom with transition to continuum and discrete states, and it also corresponds to the transmitted field from the neon atom. The OD(*ω*) is proportional to the cross-section *σ*(*ω*) (ref. 34).

### Phase determination with SPIDER

When the exponential decay time (with transition to the discrete state) is significantly longer than the IAP duration (with transition to the continuum state), the interference term [*φ*_Con_(*ω*)=*φ*_Con_(*ω*)+*ωτ*] in equation (3) can be extracted by Fourier filtering^12^,^35^. When the decay time is near the IAP duration, Fourier filtering cannot be applied and the electric-field strength (with transition to the discrete state) has to be known. Then, the absorption spectrum fitting with equations (1) and (2) is required for measured profile shown in Fig. 1c. As the Fano profile parameter *q* in equation (1) contributes to only the initial phase^1^,^11^, the value of *q* can be replaced with zero. Through the process, the electric-field strength (with transition to the discrete state) with the Lorentzian profile can be estimated. Note that when the ideal linewidth Γ of the resonance peak is much smaller than the spectrometer resolution *B* in equation (2), the resonance peak in spectral interferogram *S*(*ω*) is smeared out, which gives an over estimation of the width of the reconstructed phase. Then, the measured linewidth Γ has to be compensated by considering the spectrometer resolution *B* in equations (1) and (2).

### Temporal characterization of IAP

The IAP is characterized with the attosecond streak method^36^. In our experiment, the system configuration is similar to the above transient absorption spectroscopy. The collinearly propagated IAP and NIR pulse are focused to the helium gas (50 μm interaction length; 740 mbar backing pressure). The estimated target intensity of the NIR pulse is ~2 × 10^12^ W cm^−2^. The ionized photoelectron induced by the IAP is detected with a time-of-flight system. The resolution is 65 meV at 21 eV photoelectron energy (ionization potential of a helium atom: 24.5 eV)^23^. To reconstruct the profile and phase of the IAP, the FROG-CRAB method is used^23^.

## Author contributions

H.M. and T.Y. performed the experiments and analysed the results. K.O., A.S. and H.G. planned and coordinated the project. H.M., T.Y. and K.O. wrote the manuscript with contributions from all authors.

## Additional information

**How to cite this article:** Mashiko, H. *et al.* Characterizing inner-shell with spectral phase interferometry for direct electric-field reconstruction. *Nat. Commun.* 5:5599 doi: 10.1038/ncomms6599 (2014).
